# Prevalence Estimation Methods for Time-Dependent Antibody Kinetics of Infected and Vaccinated Individuals: A Markov Chain Approach

**DOI:** 10.1007/s11538-024-01402-0

**Published:** 2025-01-03

**Authors:** Prajakta Bedekar, Rayanne A. Luke, Anthony J. Kearsley

**Affiliations:** 1 Department of Applied Mathematics and Statistics, Johns Hopkins University, Baltimore, Maryland 21218 USA; 2 Information Technology Laboratory, National Institute of Standards and Technology, Gaithersburg, Maryland 20899 USA; 3 Department of Mathematical Sciences, George Mason University, Fairfax, Virginia 22030 USA

**Keywords:** Antibody testing, Diagnostics, Markov chain models, Prevalence estimation, Time-dependence

## Abstract

Immune events such as infection, vaccination, and a combination of the two result in distinct time-dependent antibody responses in affected individuals. These responses and event prevalence combine non-trivially to govern antibody levels sampled from a population. Time-dependence and disease prevalence pose considerable modeling challenges that need to be addressed to provide a rigorous mathematical underpinning of the underlying biology. We propose a time-inhomogeneous Markov chain model for event-to-event transitions coupled with a probabilistic framework for antibody kinetics and demonstrate its use in a setting in which individuals can be infected or vaccinated but not both. We conduct prevalence estimation via transition probability matrices using synthetic data. This approach is ideal to model sequences of infections and vaccinations, or personal trajectories in a population, making it an important first step towards a mathematical characterization of reinfection, vaccination boosting, and cross-events of infection after vaccination or vice versa.

## Introduction

As a disease becomes endemic and vaccination widespread, the ability to characterize the antibody response across time and events can become as important as the capacity to track epidemiological trends. Analysis of antibody testing can characterize the immune response to infection or vaccination and provides information with which to make population-level decisions (Caini et al. 2020; Peeling et al. 2020). Accurate interpretation of an antibody measurement randomly sampled from a population should depend on the prevalence at the time of measurement, whether the sample was obtained from a vaccinated or infected individual, and when the measurement was taken. These effects can be seen in biological literature, and have been separately modeled in cohesive probabilistic frameworks (Patrone and Kearsley 2021; Bedekar et al. 2022; Böttcher et al. 2022; Patrone et al. 2022; Luke et al. 2023). However, existing methods are not equipped to consider both transmission dynamics and time-dependent antibody response in a multiclass setting.

To overcome these real-world obstacles, we must consider mathematical models that bridge multiple scales by simultaneously considering the effects of (i) prevalence, (ii) more than two classes, and (iii) time-dependence. A shortcoming of canonical models for communicable diseases, such as susceptible-infected-recovered (SIR) models or statistical regression models, is their inability to track population-level antibody responses across time, even though many capture global transmission dynamics of viral infections by using a proxy for immune response (e.g., D’Arienzo and Coniglio 2020; McMahon et al. 2020; Quick et al. 2021; Roberto Telles et al. 2021). In one example, Dick et al. (2021) built an age-structured SIR-type model of boosting and waning immunity to severe acute respiratory syndrome coronavirus 2 (SARS-CoV-2) infection. Their model estimates prevalence as the total population with some “immunity” (not defined). To address viral evolution and antibody response, within-host differential equation models have been created, studying questions such as the protection time of antibodies (e.g., Wodarz 2005; Hernandez-Vargas and Velasco-Hernandez 2020; dePillis et al. 2023; Xu et al. 2023); such models ignore population-level trends. Finally, as an example of an approach that addresses time-dependence and antibody levels, Hay et al. (2019) applied Markov chain Monte Carlo methods to influenza infections or vaccinations in ferrets. None of these works addressed all three effects (i, ii, and iii) simultaneously, nor their complex multi-scale interactions.

There is a rich literature on longitudinal immune response dynamics (e.g., Diep et al. 2023; Guo et al. 2023; Liu et al. 2023) and many seroprevalence studies have been conducted (e.g., Osborne et al. 2000; Pollán et al. 2020; Bajema et al. 2021); a mathematical framework is needed to analyze measurements and make decisions. This is no easy feat; time-dependence, prevalence, and multiple classes combine non-trivially and pose considerable modeling challenges. In prior work we have addressed questions of time-dependence and multiclass situations separately via probabilistic modeling (Bedekar et al. 2022; Luke et al. 2023); both included prevalence estimation schemes. These works constructed mathematical machinery to calculate the probabilities of events of interest, such as single infections, but not a way to track transitions from one event to another or estimate an individual’s antibody levels associated with a sequence of events. To fully address the task of modeling real-life situations, a time-inhomogeneous Markov chain model for event-to-event transitions can be used to extend the probabilistic framework already in place.

In this paper, we combine our prior work (Bedekar et al. 2022; Luke et al. 2023) into a probabilistic model for the time-dependent antibody kinetics of the situation in which an individual either gets infected or vaccinated and then stays in that class (Sect. 3). Using methods mirroring Bedekar et al. (2022) and Luke et al. (2023), we develop a prevalence estimation scheme for naïve, infected, or vaccinated samples. We then present the same problem through the lens of a time-inhomogeneous Markov chain model (Sect. 4); such a formulation facilitates generalization to the case where reinfections and revaccinations are allowed. We demonstrate the equivalence of our extension of prior work and Markov chain frameworks by defining the transition probabilities through the class incidences and prevalence on a given time step. In addition, we develop a transition probability matrix estimation framework that allows for an equivalent method of prevalence estimation by utilizing unlabeled antibody data from the population. We validate our methods using synthetic data based on SARS-CoV-2 serological measurements (Abela et al. 2021; Congrave-Wilson et al. 2022)^1^ in Sect. 5. The discussion includes further analysis of prevalence estimation, comparisons to other approaches, limitations, and extensions (Sect. 6). We present this work to bridge the gap to the most general model in which reinfections, revaccinations, and cross events will be allowed.

## Notation

Below is a summary of baseline terminology and descriptions of terms as they pertain to our work.

### Definitions from Applied Diagnostics

- The naïve class consists of individuals who have no history of infection or vaccination. In a binary classification setting, such individuals are often referred to as ‘negative’.
- The infected class consists of individuals who have been acutely or previously infected but who are unvaccinated. In a binary classification setting, such individuals are often referred to as ‘positive’.
- The vaccinated class consists of individuals with a new or prior inoculation against a disease without a prior infection.
- Incidence refers to the fraction of new infections or vaccinations in the total population during a given time step (Bouter et al. 2023). We define an infection incidence and a vaccination incidence.
- A class prevalence during a given time step after the emergence of a disease is the fraction of individuals in the population in that class on that time step and is the sum of the incidences over all previous time steps.
- Training data correspond to sample antibody measurements from individuals for whom the true classes are known. Typically, such data are used to construct conditional probability models.
- Test data correspond to sample antibody measurements from individuals for whom the true classes are unknown or assumed to be unknown for validation purposes. Typically, a prevalence estimation procedure is applied to such data.
- Personal timeline refers to the duration since infection or vaccination for an individual.
- Absolute timeline denotes time relative to the emergence of the disease.

### Notation Specific to this Paper

- Antibody measurement is denoted by vector $\varvec{r}$. The set $\Omega$ denotes the entire measurement space.
- The prevalence for each class is denoted by $q_J$ and the incidence by $f_J$, with $J = N, I, V$ denoting the class as naïve, infected, or vaccinated. These are functions of time.
- The use of the symbol    denotes an estimated quantity.

## Multiclass Extension of Existing Time-Dependent Theory

In this section, we combine our prior work (Bedekar et al. 2022; Luke et al. 2023) to arrive at a probabilistic model for the time-dependent antibody kinetics of the situation in which an individual either gets infected or vaccinated, and then stays in that class. Here, the focus lies on explicitly enumerating all the ways in which, for example, an individual could have been infected by a certain time period. This will be contrasted with a Markov chain approach in Sect. 4 where the focus is on keeping track of transitions in the population every time period.

For both Sects. 3 and 4, the following holds true. A blood or saliva sample from an individual is measured to obtain an antibody measurement $\varvec{r}$, a vector in some compact domain $\Omega \subset {\mathbb {R}}^n$. The boundaries of $\Omega$ are governed by the measurement range of the instrument used. We generally use *t* to indicate time in the personal timeline, which is the duration since infection or vaccination for an individual. We generally use *T* to denote time in the absolute timeline in the emergence of the disease. We consider time to be discrete in this manuscript, as antibody measurements are generally reported at regular time intervals.

### Probability Models

The antibody response to infection or vaccination is time dependent, but that of an immuno-naïve individual is not. This is a basic assumption of our probability models. Let $N(\varvec{r}) = \text {Prob}(\varvec{r} | \text {Nave})$ denote the probability density that a sample yields measurement $\varvec{r}$ given that the true underlying class is naïve. Here we use Prob to denote probability density. Let $I(\varvec{r}, T) = \text {Prob}(\varvec{r}, T | \text {Infected})$ give the probability density that a sample yields measurement $\varvec{r}$ on time step *T* of the absolute timeline given that the true underlying class is infected. $V(\varvec{r}, T)$ is similarly defined for the vaccinated class. Note that in contrast to an SIR framework, we have no recovered class; the infected class consists of individuals whose symptoms may have subsided but their antibody response is still determined by the infection event. For our three classes, we assume

1 $$\begin{aligned} N:\ \Omega \rightarrow {\mathbb {R}}^+,\ \ \ I,V: \Omega \times \{0,1,2,\cdots \} \rightarrow {\mathbb {R}}^+. \end{aligned}$$

We consider time to be discretized such that one time step is of length *dt* days, as information is reported, e.g., once per day or as seven-day averages of new caseloads.

Each class has an associated prevalence: $$q_N(T), q_I(T),$$ and $$ q_V(T)$$, denoting the fraction of the population in each class at time step *T*. Prevalence quantifies the total fraction of the population incident into that class so far and thus takes values in the range [0, 1]. Since we assume that reinfection, revaccination, and infection after vaccination or vice versa do not occur, once someone is infected, they move into the infected class and stay there; similarly for vaccination. As a result, $$q_I$$ and $$q_V$$ increase over time, and $$q_N$$ decreases over time.

The probabilities above combine to form the measurement density $Q(\varvec{r}, T)$ that a sample collected on time step *T* has antibody level $\varvec{r}$. The law of total probability gives

2 $$\begin{aligned} Q(\varvec{r},T) = q_N(T) N(\varvec{r}) + q_I(T) I (\varvec{r}, T) + q_V(T) V(\varvec{r},T). \end{aligned}$$

We want to construct $I(\varvec{r}, T)$, which is naturally composed of the probabilities of being infected on different time steps before time step *T*. Via the law of total probability, following Bedekar et al. (2022), we find

3 $$\begin{aligned} I(\varvec{r}, T) = \sum _{t = 0}^T \text {Prob}(\varvec{r}, T, \text {infected on time step } t). \end{aligned}$$

Note that this conditional probability density can be determined in this straightforward way because the set of collectively exhaustive events are defined by the date of infection, since we assume this occurs once and only once. Let *R* denote the probability density of observing a measurement $\varvec{r}$, *t* time steps after infection. We then have

4 $$\begin{aligned} I(\varvec{r},T) = \sum _{t = 0}^T R(\varvec{r},t) \frac{f_I(T - t)}{q_I(T)} = \sum _{t = 0}^T R(\varvec{r}, T-t) \frac{f_I(t)}{q_I(T)}, \end{aligned}$$

where $f_I(T)$ describes the infection incidence, or fraction of the total population that is infected on time step *T* of the absolute timeline. Define $V(\varvec{r},t)$ and $f_V$ similarly, where *W* is analogous to *R*:

5 $$\begin{aligned} V(\varvec{r},T) = \sum _{t = 0}^T W(\varvec{r},t) \frac{f_V(T - t)}{q_V(T)} = \sum _{t = 0}^T W(\varvec{r}, T-t) \frac{f_V(t)}{q_V(T)}. \end{aligned}$$

Due to the assumptions stated earlier, $f_I, f_V \ge 0$. The infection and vaccination incidences sum to the prevalence of their respective classes:

6 $$\begin{aligned} q_I(T) = \sum _{t = 0}^T f_I(t), \qquad q_V(T) = \sum _{t = 0}^T f_V(t), \end{aligned}$$

and the prevalence for classes are related:

7 $$\begin{aligned} q_N(T) + q_I(T) + q_V(T) = 1. \end{aligned}$$

Motivated by the limiting behavior of antibody kinetics as in Bedekar et al. (2022), the naïve distribution is identical to both the infected and vaccinated distributions on time step 0 of the personal timeline and asymptotically:

8 $$\begin{aligned} N(\varvec{r}) = R(\varvec{r},0) = W(\varvec{r},0) = \lim _{t \rightarrow \infty } R(\varvec{r},t) = \lim _{t \rightarrow \infty } W(\varvec{r},t). \end{aligned}$$

Replacement in Eq. (2) using Eqs. (4), (5), and (8) and combining and rearranging terms gives

9 $$\begin{aligned} &  Q(\varvec{r}, T) = N(\varvec{r}) + \sum _{t = 0}^{T-1} \left[ R(\varvec{r}, T-t) - N(\varvec{r}) \right] f_I(t) \nonumber \\ &  \quad + \sum _{t = 0}^{T-1} \left[ W(\varvec{r}, T-t) - N(\varvec{r}) \right] f_V(t). \end{aligned}$$

### Prevalence Estimation

Unbiased estimators can be constructed for the prevalence of these classes, $q_N(T), q_I(T)$, and $q_V(T)$. Introduce a partition $\{D_1, D_2, D_3\}$ of the domain $\Omega$ such that

10 $$\begin{aligned} D_1 \cup D_2 \cup D_3 = \Omega \ \text { and } D_j \cap D_{\tilde{j}} = \emptyset \ \ \forall j,\tilde{j} \in \{1,2,3\} \text { such that } j \ne \tilde{j}. \end{aligned}$$

Then define

11 $$\begin{aligned} \begin{aligned} Q_j(T)&= \int _{D_j} Q(\varvec{r},T) d \varvec{r}, \quad j = 1,2,3 \\&= N_j + \sum _{t = 0}^{T-1} \left[ R_j( T-t) - N_j \right] f_I(t) + \sum _{t = 0}^{T-1} \left[ W_j( T-t) - N_j \right] f_V(t), \end{aligned} \end{aligned}$$

where

12 $$\begin{aligned} &  N_j = \int _{D_j} N(r) d \varvec{r}, \end{aligned}$$

13 $$\begin{aligned} &  R_j(T-t) = \int _{D_j} R(r, T-t) d \varvec{r}, \end{aligned}$$

and $W_j$ is defined similarly to $R_j$. Then, arbitrarily choosing to use $D_1$ and $D_2$, using Eq. (11), for $T = 1$ we have

14 $$\begin{aligned} Q_1(1) = N_1 + [R_1(1) - N_1]f_I(0) + [W_1(1) - N_1] f_V(0), \end{aligned}$$

and similarly

15 $$\begin{aligned} Q_2(1) = N_2 + [R_2(1) - N_2]f_I(0) + [W_2(1) - N_2] f_V(0). \end{aligned}$$

In matrix form this is given by

16 $$\begin{aligned} \begin{bmatrix} Q_1(1) \\ Q_2(1) \end{bmatrix} = \begin{bmatrix} R_1(1) - N_1 & W_1(1) - N_1 \\ R_2(1) - N_2 & W_2(1) - N_2 \end{bmatrix} \begin{bmatrix} f_I(0) \\ f_V(0) \end{bmatrix} + \begin{bmatrix} N_1 \\ N_2 \end{bmatrix}. \end{aligned}$$

To simplify notation, let $\varvec{f}(0) = [f_I(0), f_V(0)]^T$, $$\varvec{M}(1) = \begin{bmatrix} R_1(1) & W_1(1) \\ R_2(1) & W_2(1) \end{bmatrix}$$,

$\varvec{N}^* = [N_1, N_2]^T [1,1]$, $\varvec{N} = [N_1, N_2]^T$ and $\varvec{Q}(1) = [Q_1(1), Q_2(1)]^T$. Then $\varvec{Q}(1)$ can be written as

17 $$\begin{aligned} \varvec{Q}(1) = \left[ \varvec{M}(1) - \varvec{N}^* \right] \varvec{f}(0) + \varvec{N}. \end{aligned}$$

Solving for $\varvec{f}(0)$ gives

18 $$\begin{aligned} \varvec{f}(0) = [\varvec{M}(1) - \varvec{N}^*]^{-1} [\varvec{Q}(1) - \varvec{N}]. \end{aligned}$$

Define the Monte Carlo estimators $\hat{Q}_j(T)$ by

19 $$\begin{aligned} Q_j(T) \approx \hat{Q}_j(T) = \frac{1}{S} \sum _{\ell = 1}^S {\mathbb {I}}_j(\varvec{r}_{\ell }), \end{aligned}$$

where ${\mathbb {I}}_j$ is the indicator function on $D_j$ and $\{\varvec{r}_1, \ldots , \varvec{r}_S\}$ is the set of sample values observed on time step *T* from *S* randomly-collected samples. Let $\varvec{\hat{f}}(0) = [\hat{f}_I(0), \hat{f}_V(0)]^T$ and $\varvec{\hat{Q}}(1) = [ \hat{Q}_1(1), \hat{Q}_2(1)]^T$. Then, we can estimate $\varvec{f}(0)$, which contains the fractions of the population newly infected or vaccinated, on time step 0, via

20 $$\begin{aligned} \varvec{f}(0) \approx \varvec{\hat{f}}(0) = [\varvec{M}(1) - \varvec{N}^*]^{-1} [\varvec{\hat{Q}}(1) - \varvec{N}]. \end{aligned}$$

We will assume that the inverse in Eq. (20) exists; additional discussion follows in Sect. 4.

We now iterate in this vein to obtain linear systems in terms of previously obtained estimates. Let $\varvec{f}(t) = [f_I(t), f_V(t)]^T$, $$\varvec{M}(T-t) = \begin{bmatrix} R_1(T-t) & W_1(T-t) \\ R_2(T-t) & W_2(T-t) \end{bmatrix}$$, and $\varvec{\hat{Q}}(T) = [\hat{Q}_1(T), \hat{Q}_2(T)]^T$. We estimate the fraction of the population newly infected on time step $T-1$ to be

21 $$\begin{aligned} \hat{\varvec{f}}(T-1) = [\varvec{M}(1) - \varvec{N}^*]^{-1} \left\{ \varvec{\hat{Q}}(T) - \varvec{N} - \sum _{t = 0}^{T-2} [\varvec{M}(T-t) - \varvec{N}^*] \varvec{\hat{f}}(t) \right\} . \end{aligned}$$

Notice that we use the Monte-Carlo estimate from the population at time step *T* since the emergence of the disease to obtain information about the prevalence at the previous time step. This is because we cannot immediately discern new infections or vaccinations from the naïve population due to the time lag in personal antibody response (Bedekar et al. 2022). The estimators $\varvec{\hat{f}}(T-1)$ can be found recursively and then summed to estimate the prevalence at time step $T-1$:

22 $$\begin{aligned} \hat{\varvec{q}}(T-1) = \sum _{t = 0}^{T-1} \hat{\varvec{f}}(t), \end{aligned}$$

where the vector addition is computed element-wise and $\varvec{\hat{q}}(T-1) = [\hat{q}_I(T-1), \hat{q}_V(T-1)]^T$. Then $\hat{q}_N(T-1) = 1 - \hat{q}_I(T-1) - \hat{q}_V(T-1)$. Note that it may be easiest to fix the partition $\{D_1, D_2, D_3\}$ to compute each $\hat{\varvec{f}}(T-1)$ in the same manner. See Appendix A for a proof of the unbiasedness of the prevalence estimators, which follows from the fact that $\hat{Q}_j(\tau )$ is a Monte Carlo estimator of $Q_j(\tau )$.

## Markov Chain Approach

To begin this section, we reiterate that disallowing reinfection or revaccination significantly simplifies the task of finding $I(\varvec{r}, T)$ and $V(\varvec{r}, T)$ to a straightforward combination of our prior work (Bedekar et al. 2022; Luke et al. 2023), as given by Eqs. (4) and (5). To then apply the method, one is left with a modeling exercise to construct $R(\varvec{r}, t)$ and $W(\varvec{r},t)$ for the personal timeline antibody responses. However, if we allow reinfection and/or revaccination, the possible ways to arrive at an antibody level $\varvec{r}$ at time step *T* expand significantly, and a system that can track trajectories of infection and vaccination history becomes necessary. As an extreme example of the complexity, such a framework must be able to handle the situation in which at each next opportunity, an individual alternates between being infected and vaccinated, or repeats the same event over and over (Kocher et al. 2024). Clearly, the corresponding models for $I(\varvec{r}, T)$ and $V(\varvec{r}, T)$ are not simple constructions, as they must take all possible trajectories–the collectively exhaustive set of events of interest–into account. This section revisits the problem of no reinfection, no revaccination through a different lens, with an eye towards the setting in which reinfection, revaccination, and movement between the two classes is allowed.

A generalization of the conditional probability models $I(\varvec{r}, T)$ and $V(\varvec{r}, T)$ from Sect. 3.1 depends on transitions into infection or vaccination states, as these affect antibody level. Thus, the models should depend on a weighted sum of all potential transitions, which can be represented via a transition matrix. The population-level antibody response over time can thus be formulated in terms of the transition probabilities weighted by personal antibody response evolution. Given a current class and time *t* in personal timeline, one can compute the transition probability for the next time step. This motivates using a discrete-time Markov chain framework, because only the current state (*N*, *I*, *V*) and conditions $(\varvec{r}, t, T)$ affect the next state. To consider the event of infection separately from previous infection, we partition class *I* from Sect. 3.1 into two states representing new infections ($\mathcal {I}$) and previous infections ($\mathcal {I}'$). Similarly, we partition *V* into $\mathcal {V}$ and $\mathcal {V}'$.

### Transition Probabilities

A transition matrix *S* defines the probabilities of moving between states. Here, *S*(*i*, *j*) is the probability of moving *to* state *i* *from* state *j*, where the ordering is $N, \mathcal {I}', \mathcal {I}, \mathcal {V}', \mathcal {V}$. Let $T =0$ index the emergence of a disease. We assume that our initial state vector is $X_{-1} = \varvec{e}_1$ to model the disease emergence, so that everyone is in state *N* with probability 1 on the day before the disease emerges. Here, $\varvec{e}_1$ is the first unit vector^2^. Let $X_j$ denote the state, or class, at time step *j*. We disallow transition from $\mathcal {I}'$ or $\mathcal {V}'$ back to *N*, which is reasonable for a time scale on the order of several months. Denote the transition probabilities by *s*.

**Fig. 1:**
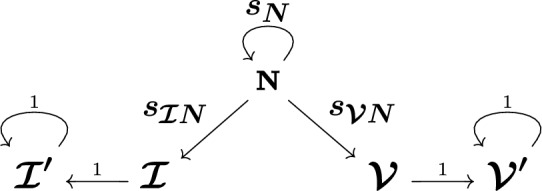
Graph describing the allowable movements between states. Here, *N* is naïve, $\mathcal {I}$ is newly infected, $\mathcal {I}'$ is previously infected, $\mathcal {V}$ is newly vaccinated, and $\mathcal {V}'$ is previously vaccinated. Double subscripts on *s* denote the transition probability from the second state to the first

We employ a graph to represent our framework, in which each state or class is a node and transitions between classes are directed, weighted edges; see Fig. 1. We let $s_N(T)$ denote the weight of the degenerate edge to *N* from *N*, or the probability of staying naïve. The probabilities $s_{\mathcal {I}N}(T)$ and $s_{\mathcal {V}N}(T)$ weight the edges to $\mathcal {I}$ from *N* and to $\mathcal {V}$ from *N*, indicating infection or vaccination, respectively. Since we forbid reinfection or revaccination, one moves to $\mathcal {I}'$ from $\mathcal {I}$ or to $\mathcal {V}'$ from $\mathcal {V}$ on the next time step with probability 1. Once in $\mathcal {I}'$ or $\mathcal {V}'$, one remains there with probability 1 and mounts their antibody response.

By definition, the transition probabilities depend on $f_I(T)$ and $f_V(T)$, the fractions of the population that are infected and vaccinated on time step *T*, respectively. At time step *T*, the fraction of the population becoming newly infected or vaccinated is divided by the relative size of the naïve population at the previous step to obtain the transition probability. This assumes that, of all the individuals in the naïve class on the previous time step, a percentage $f_\mathcal {I}$ or $f_\mathcal {V}$ move to $\mathcal {I}$ and $\mathcal {V}$, respectively. Thus, we have

23a $$\begin{aligned} s_{\mathcal {I}N}(T) = \frac{f_I(T)}{q_N(T-1)}, \end{aligned}$$

23b $$\begin{aligned} s_{\mathcal {V}N}(T) = \frac{f_V(T)}{q_N(T-1)}, \end{aligned}$$

23c $$\begin{aligned} s_N(T) = 1 - s_{\mathcal {I}N}(T) - s_{\mathcal {V}N}(T). \end{aligned}$$

The final relationship between the transition rates, the one defining $s_N(T)$, is a total probability statement. Here we define $q_N(-1) = 1$ to be consistent with our assumption that everyone is in the naïve class on the day before the disease emerges. When $q_N(T-1) = 0$ we define $s_{\mathcal {I}N}(T)$ and $s_{\mathcal {V}N}(T)$ to be zero, as there are no individuals remaining in the *N* class, so transitions out of that class are impossible.

We note a difference between the transition probabilities and the quantity *f*(*t*)/*q*(*T*) in Bedekar et al. (2022) as well as in Eqs. (4) and (5). In the context of these probabilistic models, this ratio *f*(*t*)/*q*(*T*) is the proportion of those who became infected on time step *t* of the disease emergence ($t<T$) out of the entire previously infected population as of time step *T*, and it is used to write out the total probability equation for time step *T*. In contrast, the transition probabilities $s_{\mathcal {I}N}(T)$ and $s_{\mathcal {V}N}(T)$ denote the fraction of the population that are newly infected or vaccinated *out of those available*; i.e., the proportion of the population that was naïve the time step before.

The transition matrix for movement from time step $T-1$ to time step *T* is thus given by

24 $$\begin{aligned} S(T) = \begin{bmatrix} 1 - s_{\mathcal {I}N}(T) - s_{\mathcal {V}N}(T) & 0 & 0 & 0 & 0 \\ 0 & 1 & 1 & 0 & 0 \\ \frac{f_I(T)}{q_N(T-1)} & 0 & 0 & 0 & 0 \\ 0 & 0 & 0 & 1 & 1 \\ \frac{f_V(T)}{q_N(T-1)} & 0 & 0 & 0 & 0 \end{bmatrix}, \end{aligned}$$

where the ordering is $N, \mathcal {I}', \mathcal {I}, \mathcal {V}', \mathcal {V}$. As an example, we distill the probability of a person ending up in a particular class after three time steps using product of transition matrices as follows:

25 $$\begin{aligned} S(2)S(1)S(0)\textbf{e}_1 = \begin{bmatrix} s_N(2)s_N(1)s_N(0) \\ s_{\mathcal {I}N}(0) + s_{\mathcal {I}N}(1) s_N(0) \\ s_{\mathcal {I}N}(2) s_N(1) s_N(0) \\ s_{\mathcal {V}N}(0) + s_{\mathcal {V}N}(1) s_N(0) \\ s_{\mathcal {V}N}(2) s_N(1) s_N(0) \end{bmatrix}. \end{aligned}$$

The interpretation of the first three entries of the resulting vector is as follows: the probability that one stays naïve through time step 3; the probability that one got infected on time step 1 and is considered previously infected on time step 2 plus the probability that one got infected on time step 2 and is considered previously infected on time step 3; and the probability that one did not get infected on time steps 1 or 2 but did get infected on time step 3. In short, we confirm that all possible trajectories to *N*, $\mathcal {I}'$, and $\mathcal {I}$ on time step 3 are considered. Parallel interpretations hold for $\mathcal {V}'$ and $\mathcal {V}$ as for $\mathcal {I}'$ and $\mathcal {I}$.

Specifically, such multi-step transitions can be represented in terms of the entries of the matrix–vector product of the subsequent transitions. A $\tau$ step transition from time step 0 to time step $\tau$ is represented by $H_\tau ,$

26 $$\begin{aligned} H_\tau = S(\tau ) S(\tau -1)\cdots S(1)S(0) = \left( \prod _{t = 0}^{\tau } S(\tau - t) \right) , \end{aligned}$$

where $\tau - t$ is used to ensure indexing of the product in the correct order. Thus, if everyone starts in the naïve class on time step 0, then the total probabilistic distribution of the classes after *T* time steps in the absolute timeline will be

27 $$\begin{aligned} \begin{bmatrix} \text {Prob}(X_{T} = N) \\ \text {Prob}(X_{T} = \mathcal {I}')\\ \text {Prob}(X_{T} = \mathcal {I}) \\ \text {Prob}(X_{T} = \mathcal {V}') \\ \text {Prob}(X_{T} = \mathcal {V}) \end{bmatrix} = H_T \varvec{e}_1. \end{aligned}$$

### Equivalence of State Probabilities and Original Framework Prevalence

Here, we continue to assume that everyone begins in the naïve class at time step zero, or that our initial state vector is $\varvec{e}_1$. By using Eq. (27), the state probabilities are given explicitly by

28a $$\begin{aligned} \text {Prob}(X_T = N)&= \prod _{t = 0}^{T} s_N(t), \end{aligned}$$

28b $$\begin{aligned} \text {Prob}(X_T = \mathcal {I}')&= \sum _{t = 0}^{T-1} s_{\mathcal {I}N}(t) \prod _{\tau = 0}^{t-1} s_N(\tau ), \end{aligned}$$

28c $$\begin{aligned} \text {Prob}(X_T = \mathcal {I})&= s_{\mathcal {I}N}(T) \prod _{t = 0}^{T-1} s_N(t), \end{aligned}$$

28d $$\begin{aligned} \text {Prob}(X_T = \mathcal {V}')&= \sum _{t = 0}^{T-1} s_{\mathcal {V}N}(t) \prod _{\tau = 0}^{t-1} s_N(\tau ), \end{aligned}$$

28e $$\begin{aligned} \text {Prob}(X_T = \mathcal {V})&= s_{\mathcal {V}N}(T) \prod _{t = 0}^{T-1} s_N(t). \end{aligned}$$

The state probabilities and the prevalence in Sect. 3.2 are related; in fact, $\textrm{Prob} (X_T = N) = q_N(T), \textrm{Prob} (X_T = \mathcal {I}') = q_I(T-1), \textrm{Prob} (X_T = \mathcal {I}) = f_I(T),$ $\textrm{Prob} (X_T = \mathcal {V}') = q_V(T-1),$ and $\textrm{Prob} (X_T = \mathcal {V}) = f_V(T)$, as proved below. Using the definition of the state probability and Eq. (23), we have

29 $$\begin{aligned} \begin{aligned} \textrm{Prob} (X_{T} = N)&= \prod _{t = 0}^{T} s_N(t) = \prod _{t = 0}^{T} \left[ 1 - s_{\mathcal {I}N}(t) - s_{\mathcal {V}N}(t) \right] \\&= \prod _{t = 0}^{T} \left[ 1 - \frac{f_I(t)}{q_N(t-1)} - \frac{f_V(t)}{q_N(t-1)} \right] . \end{aligned} \end{aligned}$$

Using Eqs. (6) and (7) and the fact that $q_N(0) = 1$, we then find

30 $$\begin{aligned} \begin{aligned} \textrm{Prob} (X_{T} = N)&= \prod _{t = 0}^{T} \left[ \frac{q_N(t-1) - f_I(t) - f_V(t)}{q_N(t-1)} \right] \\&= \prod _{t = 0}^{T} \left[ \frac{1 - q_I(t-1) - q_V(t-1) - f_I(t) - f_V(t)}{q_N(t-1)} \right] \\&= \prod _{t = 0}^{T} \left[ \frac{1 - q_I(t) - q_V(t)}{q_N(t-1)} \right] \\&= \prod _{t = 0}^{T} \frac{ q_N(t)}{q_N(t-1)} = q_N(T). \end{aligned} \end{aligned}$$

Using Eq. (30), we find that for $\mathcal {I}'$,

31 $$\begin{aligned} \textrm{Prob} (X_{T} = \mathcal {I}')= &  \sum _{t = 0}^{T-1} s_{\mathcal {I}N} (t) \prod _{\tau = 0}^{t-1} s_N(\tau ) \nonumber \\ = &  \sum _{t = 0}^{T-1} \frac{f_I(t)}{q_N(t-1)} q_N(t-1) = \sum _{t = 0}^{T-1} f_I(t) = q_I(T-1). \end{aligned}$$

This is expected, because the previously infected class does not include new infections occurring on time step *T*. Finally, using Eq. (30), for $\mathcal {I}$ we find

32 $$\begin{aligned} \begin{aligned} \textrm{Prob}(X_T = \mathcal {I})&= s_{\mathcal {I}N}(T) \prod _{t = 0}^{T-1} s_N(t) = s_{\mathcal {I}N}(T) q_N(T-1) \\&= \frac{f_I(T)}{q_N(T-1)} q_N(T-1) = f_I(T). \end{aligned} \end{aligned}$$

One can analogously show that $\textrm{Prob} (X_T = \mathcal {V}') = q_V(T-1)$ and $\textrm{Prob} (X_T = \mathcal {V}) = f_V(T)$. Thus,

33 $$\begin{aligned} &  \text {Prob} (X_T = N) + \text {Prob} (X_T = \mathcal {I}') + \text {Prob} (X_T = \mathcal {I}) \nonumber \\  &  \qquad + \text {Prob} (X_T = \mathcal {V}') + \text {Prob} (X_T = \mathcal {V}) \nonumber \\  &  \qquad \qquad = q_N(T) + q_I(T-1) + f_I(T) + q_V(T-1) + f_V(T) \nonumber \\  &  \qquad \qquad = q_N(T) + q_I(T) + q_V(T) = 1. \end{aligned}$$

Since we have split the infected class of Sect. 3 into $\mathcal {I}$ and $\mathcal {I}'$ to represent new and old infections, we expect that $\textrm{Prob} (X_T = \mathcal {I}') + \textrm{Prob} (X_T = \mathcal {I}) = q_I(T)$; this is in fact true, as $q_I(T-1) + f_I(T) = q_I(T)$. This allows us to confirm the relationship between the state probabilities and the prevalence of Sect. 3.2 and that the state probabilities sum to 1, as expected.

Equations (30), (31), (32) and analogous statements for $\mathcal {V}', \mathcal {V}$ help rewrite the $\tau -$ step transition matrix $H_\tau$ as follows:

34 $$\begin{aligned} H_{\tau } = \begin{bmatrix} q_N(\tau ) & 0 & 0 & 0 & 0\\ \sum \limits _{t=0}^{\tau -1} f_I(t) & 1 & 1 & 0 & 0\\ f_I(\tau ) & 0 & 0 & 0 & 0\\ \sum \limits _{t=0}^{\tau -1} f_V(t) & 0 & 0 & 1 & 1\\ f_V(\tau ) & 0 & 0 & 0 & 0 \end{bmatrix}, \end{aligned}$$

where the ordering is $N, \mathcal {I}', \mathcal {I}, \mathcal {V}', \mathcal {V}$. Notice that these transitions are as expected. However, these transition matrices do not carry over information about antibody kinetics, nor the convolution between personal and absolute timelines that leads to a given sampled antibody value from a population on a given time. We will incorporate this in the next subsection.

### Conditional Probabilities in Terms of Transition Matrices and Personal Timeline Models

The set of previously infected individuals can be partitioned using the time period when they were newly infected, i.e., when they were in the transient class $\mathcal {I}'$. Thus, using the law of total probability, the conditional probability density for an antibody measurement $\varvec{r}$ during time step *T* in the absolute timeline given that the sample comes from a previously infected individual is

35 $$\begin{aligned} \begin{aligned} \text {Prob}(\varvec{r},T | X_T = \mathcal {I}')&= \sum \limits _{t=0}^{T-1} \text {Prob}(\varvec{r},T, X_{t} = \mathcal {I}| X_T = \mathcal {I}') \\&= \frac{1}{\text {Prob}(X_T = \mathcal {I}')}\sum \limits _{t=0}^{T-1} \text {Prob}(\varvec{r},T, X_{t} = \mathcal {I}, X_T = \mathcal {I}'). \end{aligned} \end{aligned}$$

This summand consists of people who were naïve through time period $t-1$, become newly infected in time period *t*, and stay in the previously infected class thereafter, i.e. $NN\cdots N \mathcal {I}\mathcal {I}'\mathcal {I}'\cdots \mathcal {I}'$. For such a sequence, $R(\varvec{r},T-t)$ is the distribution of the antibody response on time step *T* in the absolute timeline. Thus,

36 $$\begin{aligned} \begin{aligned} \text {Prob}(\varvec{r},T, X_{t} = \mathcal {I}, X_T = \mathcal {I}')&= R(\varvec{r},T - t) s_{\mathcal {I}N}(t) \prod _{\tau = 0}^{t-1} s_N(\tau ) \\&= R(\varvec{r},T - t) \left<\left( \prod _{\tau = 0}^{t} S(t-\tau ) \right) \varvec{e}_1, \varvec{e}_3\right>. \end{aligned} \end{aligned}$$

Here, angle brackets denote the (dot) inner product. In total, the conditional probability density can be rewritten as

37 $$\begin{aligned} \begin{aligned} \text {Prob}(\varvec{r},T | X_T = \mathcal {I}')&= \frac{1}{\text {Prob}(X_T = \mathcal {I}')} \left( \sum _{t = 0}^{T-1} R(r,T - t) \left<\left( \prod _{\tau = 0}^{t} S(t-\tau ) \right) \varvec{e}_1, \varvec{e}_3\right> \right) \\&= \frac{1}{\left<H_T \varvec{e}_1, \varvec{e}_2\right>} \left( \sum _{t = 0}^{T-1} R(r,T - t) \left<H_t \varvec{e}_1, \varvec{e}_3\right> \right) .\\ \end{aligned} \end{aligned}$$

In other words, the inner product inside the large parentheses is the prevalence of newly infected individuals on a particular time step. Thus, the sum in the last term is the inner product of responses on different time steps with the vector of newly infected prevalence. Using Eq. (28b)-(28c), we can see that this is solely in terms of the transition matrix *S* and the personal antibody response model *R*. The expression for $\textrm{Prob} (\varvec{r}, T | X_T = \mathcal {V}')$ is analogous.

The expression for $\textrm{Prob} (\varvec{r}, T | X_T = \mathcal {I})$ is simpler than that for $\mathcal {I}'$ because there is only one possible sequence of state transitions: $N N \cdots N \mathcal {I}$, where the transition from *N* to $\mathcal {I}$ occurs on time step *T*. This sequence has antibody response distribution $R(\varvec{r}, 0 ) = N(\varvec{r})$, and thus $\textrm{Prob} (\varvec{r}, T | X_T = \mathcal {I}) = \textrm{Prob} (\varvec{r}, T | X_{T-1} = N, X_T = \mathcal {I}) = N(\varvec{r})$.

#### Equivalence of Measurement Density in Markov Chain and Original Frameworks

Ideas similar to Eq. (2) lead us to derive the measurement density,

38 $$\begin{aligned} \begin{aligned} Q(\varvec{r}, T)&= q_N(T) \textrm{Prob} (\varvec{r}, T | X_T = N) \\&\qquad + q_{\mathcal {I}'}(T) \textrm{Prob} (\varvec{r}, T | X_T = \mathcal {I}') + q_{\mathcal {I}}(T) \textrm{Prob} (\varvec{r}, T | X_T = \mathcal {I}) \\&\qquad + q_{\mathcal {V}'}(T) \textrm{Prob} (\varvec{r}, T | X_T = \mathcal {V}') + q_{\mathcal {V}}(T) \textrm{Prob} (\varvec{r}, T | X_T = \mathcal {V}). \end{aligned} \end{aligned}$$

Using Eqs. (28) and (37), the relationship between the state probabilities and the prevalence in Sect. 3.2, and the above discussion, after some rearranging, Eq. (38) can be written as

39 $$\begin{aligned} \begin{aligned} Q(\varvec{r},T)&= q_N(T-1) N(\varvec{r}) + \sum _{t = 0}^{T-1} R(\varvec{r},T - t) \left<\left( \prod _{\tau = 0}^{t} S(t-\tau ) \right) \varvec{e}_1, \varvec{e}_3\right> \\&+ \sum _{t = 0}^{T-1} W(\varvec{r},T - t) \left<\left( \prod _{\tau = 0}^{t} S(t-\tau ) \right) \varvec{e}_1, \varvec{e}_5\right> . \end{aligned} \end{aligned}$$

Here we have also used $q_N(T-1) = q_N(T) + f_I(T) + f_V(T)$. While Eq. (39) is a different representation of the measurement density from that given by Eq. (9) in Sect. 3, by rewriting the inner products as state probabilities and using the relationship between the state probabilities and the prevalence in Sect. 3.2, one can show their equivalence:

40 $$\begin{aligned} \begin{aligned} Q(\varvec{r},T)&= \left[ 1 - q_I(T-1) - q_V(T-1) \right] N(\varvec{r}) \\&+ \sum _{t = 0}^{T-1} R(\varvec{r},T - t) \text {Prob}(X_t = \mathcal {I}) + \sum _{t = 0}^{T-1} W(\varvec{r},T - t) \text {Prob}(X_t = \mathcal {V}) \\&= \left[ 1 - \sum _{t = 0}^{T-1} f_I(t) - \sum _{t = 0}^{T-1} f_V(t) \right] N(\varvec{r}) \\&\qquad + \sum _{t = 0}^{T-1} R(\varvec{r},T - t) f_I(t) + \sum _{t = 0}^{T-1} W(\varvec{r},T - t) f_V(t) \\&= N(\varvec{r}) + \sum _{t = 0}^{T-1} [R(\varvec{r},T - t) - N(\varvec{r}) ]f_I(t) + \sum _{t = 0}^{T-1} [W(\varvec{r},T - t) - N(\varvec{r})] f_V(t). \end{aligned} \end{aligned}$$

Due to this equivalence, prevalence estimation in this Markov chain approach will follow Sect. 3.2. Then, original class *I* will be broken into $\mathcal {I}$ and $\mathcal {I}'$ so that the estimators are $\hat{q}_{\mathcal {I}'}(T) = \sum _{t = 0}^{T-1} \hat{f}_I(t)$ and $\hat{q}_{\mathcal {I}}(T) = \hat{f}_I(T)$ by their definitions. Similar estimators can be found for $q_{\mathcal {V}'}(T)$ and $q_{\mathcal {V}}(T)$.

### Estimation of Transition Probability Matrices

Let us reconsider the measurement density Eq. (39), from Sect. 4.3.1. We note that it can be written in terms of the transition matrix $H_t$ from Eq. (34) as

41 $$\begin{aligned} \begin{aligned} Q(\varvec{r},T)&= q_N(T-1) N(\varvec{r}) + \sum _{t = 0}^{T-1} R(\varvec{r},T - t) H_{t,(3,1)} + \sum _{t = 0}^{T-1} W(\varvec{r},T - t) H_{t,(5,1)}, \end{aligned} \end{aligned}$$

where $H_{t,(k,1)}:= \langle H_t \varvec{e}_1, \varvec{e}_k\rangle .$ Integrating both sides of the equation over some subdomain $D_j$ of the antibody measurement space leads to

42 $$\begin{aligned} \begin{aligned} Q_{D_j}(T)&= q_N(T-1) N_{D_j} + \sum _{t = 0}^{T-1} R_{D_j}(T - t) H_{t,(3,1)} + \sum _{t = 0}^{T-1} W_{D_j}(T - t) H_{t,(5,1)}. \end{aligned} \end{aligned}$$

Unbiased estimation of $Q_{D_j}$ can be achieved by Monte-Carlo estimation as in Sect. 3.2: by measuring antibody levels for randomly selected samples from the population during time period *T*, followed by a counting of the fraction of the measurements that fall in that subdomain.

Notice that for $T = 0,$ we have $Q_{D_j}(0) = N_{D_j}$; this follows our assumption that every person starts naïve at $T = -1$, the day before the disease emerges, and that antibody response does not mount immediately after infection. That is, the total probability mass is distributed exactly as the naïve distribution: this is as expected. For $T=1$,

43 $$\begin{aligned} Q_{D_j}(1) = q_N(0) N_{D_j} + R_{D_j}(1) H_{0,(3,1)} + W_{D_j}(1) H_{0,(5,1)}. \end{aligned}$$

Let $D_1, D_2, D_3$ partition the domain as in Eq. (10). Notice that for such a partition, as *N*, *R*(*t*), *W*(*t*) are probability distributions for all $t\ge 0$,

44 $$\begin{aligned} \begin{aligned} N_{D_1} + N_{D_2} + N_{D_3}&= R_{D_1}(t) + R_{D_2}(t) + R_{D_3}(t) = W_{D_1}(t) + W_{D_2}(t) + W_{D_3}(t) \\&= 1 = Q_{D_1}(t) + Q_{D_2}(t) + Q_{D_3}(t). \end{aligned} \end{aligned}$$

Using Eq. (43) for $D_1, D_2, D_3$, we can write down a system of linear equations, given by

45 $$\begin{aligned} \begin{bmatrix} N_{D_1} & R_{D_1}(1) & W_{D_1}(1)\\ N_{D_2} & R_{D_2}(1) & W_{D_2}(1)\\ 1 & 1 & 1 \end{bmatrix} \begin{bmatrix} q_N(0) \\ H_{0,(3,1)}\\ H_{0,(5,1)} \end{bmatrix} = \begin{bmatrix} Q_{D_1}(1) \\ Q_{D_2}(1)\\ q_N(-1) \end{bmatrix} \approx \begin{bmatrix} \widehat{Q}_{D_1}(1) \\ \widehat{Q}_{D_2}(1)\\ q_N(-1) \end{bmatrix} \end{aligned}$$

where the term $q_N(-1)=1$ as before due to our assumption that everyone starts naïve before the emergence of the disease. The last equation of this matrix system arises out of an application of Eq. (44), and expresses that the naïve population in the preceding time step distributes into naïve, newly infected, and newly vaccinated in the next time step.

We can thus estimate $q_N(0), H_{0,(3,1)}, H_{0,(5,1)}$ and obtain the respective estimates $\widehat{q_N}(0), \widehat{H}_{0,(3,1)},$ and $\widehat{H}_{0,(5,1)}$. Via induction, for a general *T* we obtain the following system

46 $$\begin{aligned} &  \begin{bmatrix} N_{D_1} & R_{D_1}(1) & W_{D_1}(1)\\ N_{D_2} & R_{D_2}(1) & W_{D_2}(1)\\ 1 & 1 & 1 \end{bmatrix} \begin{bmatrix} q_N(T-1) \\ H_{(T-1)(3,1)}\\ H_{(T-1)(5,1)} \end{bmatrix}\nonumber \\ &  \quad = \begin{bmatrix} Q_{D_1}(T) - \sum \limits _{t=0}^{T-2} \left( R_{D_1}(T-t) H_{t,(3,1)} + W_{D_1}(T-t) H_{t,(5,1)} \right) \\ Q_{D_2}(T) - \sum \limits _{t=0}^{T-2} \left( R_{D_2}(T-t) H_{t,(3,1)} + W_{D_2}(T-t) H_{t,(5,1)} \right) \\ q_N(T-2) \end{bmatrix} \nonumber \\ &  \quad \approx \begin{bmatrix} \widehat{Q}_{D_1}(T) - \sum \limits _{t=0}^{T-2} \left( R_{D_1}(T-t) \widehat{H}_{t,(3,1)} + W_{D_1}(T-t) \widehat{H}_{t,(5,1)} \right) \\ \widehat{Q}_{D_2}(T) - \sum \limits _{t=0}^{T-2} \left( R_{D_2}(T-t) \widehat{H}_{t,(3,1)} + W_{D_2}(T-t) \widehat{H}_{t,(5,1)} \right) \\ \widehat{q}_N(T-2) \end{bmatrix}. \end{aligned}$$

Note that even though we only estimate values pertaining to the naïve, newly infected, and newly vaccinated entry in the first column, these sequential estimates determine the values for previously infected and vaccinated entries by the following recursive relation:

47 $$\begin{aligned} \begin{aligned} H_{(T-1)(2,1)}&= H_{(T-2)(2,1)} + H_{(T-2)(3,1)}\\&= H_{(T-3)(2,1)} + H_{(T-3)(3,1)} + H_{(T-2)(3,1)} = \cdots \\&= H_{0(2,1)} + \sum \limits _{\tau =0}^{T-2} H_{\tau (3,1)}\\&= \sum \limits _{\tau =0}^{T-2} H_{\tau (3,1)} \approx \sum \limits _{\tau =0}^{T-2} \widehat{H}_{\tau (3,1)}. \end{aligned} \end{aligned}$$

and similarly

48 $$\begin{aligned} \begin{aligned} H_{(T-1)(4,1)}&= H_{(T-2)(4,1)} + H_{(T-2)(5,1)} \\&\hspace{2mm} \vdots \\&= H_{0(4,1)}+\sum \limits _{\tau =0}^{T-2} H_{\tau (5,1)} = \sum \limits _{\tau =0}^{T-2} H_{\tau (5,1)} \approx \sum \limits _{\tau =0}^{T-2} \widehat{H}_{\tau (5,1)}. \end{aligned} \end{aligned}$$

This is as expected because the prevalence of previously infected/vaccinated is obtained as an accumulation of prevalence for newly infected/vaccinated over the entire absolute timeline.

## Example Applied to SARS-CoV-2 Antibody Data

In the context of SARS-CoV-2, our models characterize the time frame around spring 2021, when individuals either had a previous infection or were receiving their first vaccination, with very few people having done both. We create synthetic training data motivated by clinical data from Abela et al. (2021) and Congrave-Wilson et al. (2022). The synthetic data are created by studying immunoglobulin G (IgG) measurements for naïve, infected, and vaccinated individuals. These values are considered together to have arbitrary units (AU) and log-transformed similarly to Patrone and Kearsley (2021), Bedekar et al. (2022), and Luke et al. (2023) to yield the unit-less, one-dimensional measurement

49 $$\begin{aligned} r = \log _2(\tilde{r}). \end{aligned}$$

We use gamma distributions to model the infected and vaccinated antibody responses *t* days after infection or vaccination, which both change with time, and the naïve distribution. We use pre-vaccine measurements reported as SARS-CoV-2-naïve to model the naïve population with

50 $$\begin{aligned} N(r) = \frac{1}{\Gamma (\alpha _n) \beta _n^{a_n}} r^{\alpha _n-1} e^{-r/\beta _n}. \end{aligned}$$

For this synthetic dataset, $\alpha _n = 15.1$ and $\beta _n = 0.184$. We allow $\alpha$ to vary in time for both the vaccinated and infected responses as

51 $$\begin{aligned} \alpha _c(t) = \frac{\theta _{1,c} t}{1 + \theta _{2,c} t^2} + \alpha _n, \text { where } c \in \{i,v\}, \end{aligned}$$

where the subscripts *i* and *v* denote infected and vaccinated. The model for the personal timeline of an infected individual is then given by

52 $$\begin{aligned} R(r,t) = \frac{1}{\Gamma (\alpha (t)) \beta _n^{a(t)}} r^{\alpha (t)-1} e^{-r/\beta _n}. \end{aligned}$$

**Fig. 2:**
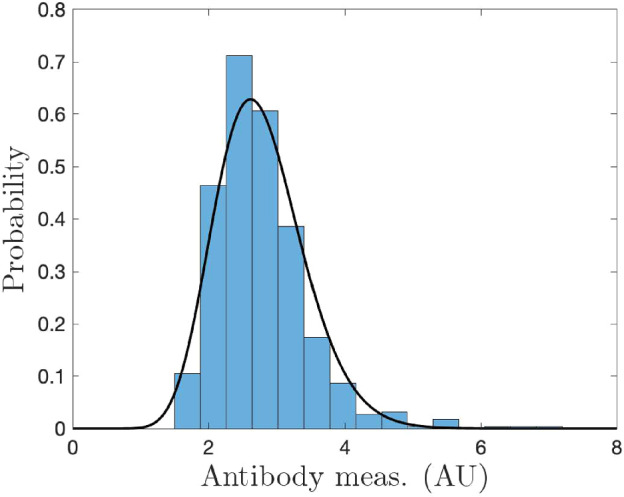
Log-transformed synthetic antibody measurements from the naïve population with corresponding probability model (Color Figure Online)

**Fig. 3:**
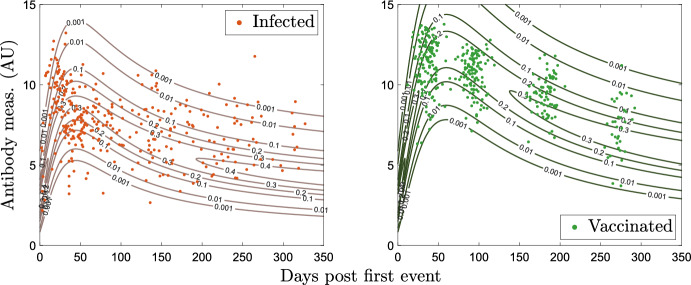
Log-transformed synthetic antibody measurements from the infected and vaccinated populations with corresponding probability models (Color Figure Online)

Following Bedekar et al. (2022), we require that at $t = 0$, both the vaccinated and infected models are identical to *N*(*r*). This is enforced by our modeling; note that the shape and scale at $t=0$ and as $t\rightarrow \infty$ are identical to those for naïve. The model for personal timeline for vaccination, *W*(*r*, *t*), is given similarly to Eq. (52). The model parameters are $\theta _{1, i} = 1.56, \theta _{2, i} = 5.1 \times 10^{-4}, \theta _{1, v} = 1.74$, and $\theta _{2, v} = 2.8 \times 10^{-4}$. The models are shown in Figs. 2 and 3.

### Prevalence Estimation Via Transition Probability Matrices

We conduct prevalence estimation for test data sampled from the probability models described above and through transition probability matrix estimation methods developed in Sect. 4.4. We create 1000 sets of synthetic test data for various numbers of sample points $N_s$ to calculate the mean and standard deviation of the prevalence estimates. We discretize time into time steps of $dt = 21$ days and use 10 time periods. This results in 210 days total, or about 7 months, which is on the order of the synthetic training data.

To mimic a wave of infections during the emergence of a disease, we assume a sinusoidal change in the infected prevalence per time period, given by

53 $$\begin{aligned} f_I(0) = 0.01, \quad f_I(t) = 0.01 \sin \left( \frac{\pi t}{10} \right) , \quad t \in \{1, \ldots , 10\}. \end{aligned}$$

This gives the corresponding true newly plus previously infected prevalence as

54 $$\begin{aligned} q_I(t) = 0.01 \left[ 1 + \sum _{\tau = 0}^t \sin \left( \frac{ \pi \tau }{10} \right) \right] , \quad t \in \{0, 1, \ldots , 10\}. \end{aligned}$$

We also assume a constant rate of vaccination, given by an incidence

55 $$\begin{aligned} f_V(t) = 0.01, \quad t \in \{0, 1, \ldots , 10\}, \end{aligned}$$

and corresponding true newly and previously vaccinated prevalence as

56 $$\begin{aligned} q_V(t) = 0.01 t + 0.01, \quad t \in \{0, 1, \ldots , 10\}. \end{aligned}$$

Thus, the prevalence of naïve for time-period *t* is

57 $$\begin{aligned} q_N(t) = 1 - q_V(t) - q_I(t) = 0.08 - 0.01 t -0.01 \sum _{\tau = 0}^t \sin \left( \frac{ \pi \tau }{10} \right) , \quad t \in \{0, 1, \ldots , 10\}.\nonumber \\ \end{aligned}$$

These incidence rates lead to the true one-step and $\tau$-step transition matrices by using Eqs. (24) and (34) respectively. The $\tau$-step transition matrix entries are then estimated using methods detailed in Sect. 4.4, which provide us with estimates of prevalence for different classes.

**Fig. 4:**
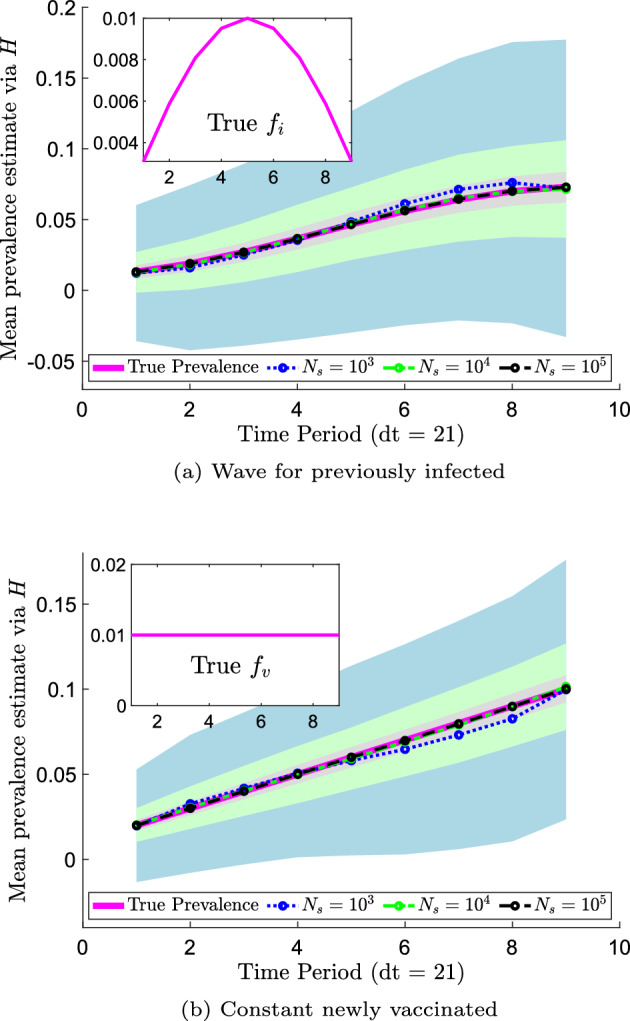
Prevalence estimation via transition probability estimates using synthetic data for antibody responses. The mean over 1000 synthetic data sets is shown for various numbers of samples $N_s$ with standard deviation confidence intervals (shown in a lighter shade of the corresponding color of $N_s$) over time (Color Figure Online)

The results of prevalence estimation are shown in Fig. 4. The mean prevalence estimates are shown as data points and corresponding standard deviations are shown as shaded regions in lighter colors. The mean estimates agree fairly well across the sample sizes and with the true values in both the infected and vaccinated cases. We note that for low sample sizes, such as $N_s = 10^3$, some prevalence estimates are negative, which are infeasible. However, the standard deviation of the estimates decreases with increasing sample size as expected. We observe larger prevalence estimation standard deviations at later time periods, as expected following Bedekar et al. (2022), who noted that errors accumulate over time. For $N_s = 10^5$, the average prevalence estimate errors across all time periods are $(17 \pm 15)$ % for infected and $(8.6 \pm 7.0)$ % for vaccinated.

The results of prevalence estimation via the methods developed in Sect. 3.2 (not shown) are essentially identical: the norms of the differences, taken across all time periods, of the means and standard deviations produced by the two methods are less than $1.5 \times 10^{-14}$ for both *I* and *V*. This is as expected, as we have shown in Sect. 4.3.1 that the measurement densities under these two frameworks are equivalent.

## Discussion

We have shown the equivalence of the probabilistic modeling approaches in Bedekar et al. (2022) and Luke et al. (2023) and the time-inhomogeneous Markov chain approach for the case of multiclass time-dependent effects that preclude reinfection, revaccination, or any cross-effects. Useful immediately after the introduction of vaccines, this case is restrictive for general use. The equivalence itself is a significant contribution as we anticipate that our Markov chain approach will be effective in modeling the most general case. As a very promising result, prevalence estimation via transition probability matrices is identical in expectation to that of the multiclass extension of the existing time-dependent framework (Sect. 3); the former can reliably be used as we address the most general version of the problem.

### More on Prevalence Estimation

We begin with a few comments on prevalence estimation. In Sect. 3.1, we noted how we discretize time in time steps of *dt* days to follow batched reporting trends. Daily measurements are rarely available and testing delays occur; using a larger value of *dt* provides less detailed information, but results in better prevalence estimates when data are sparse (Bedekar et al. 2022). We also note that for prevalence estimation via transition probability matrices, the system given by Eq. (45) is invertible provided the choice of time step *dt* and subdomain partition is such that $R_{D_j}(1), W_{D_j}(1), \text { and } N_{D_j}$ are well separated from each other. This ensures that the antibody response of infected or vaccinated individuals is separable from that of naïve population. Further, a careful selection of subdomains $D_j$ as guided by Patrone and Kearsley (2024), Luke et al. (2023), and Bedekar et al. (2022) can help the estimation by minimizing numerical errors.

**Fig. 5:**
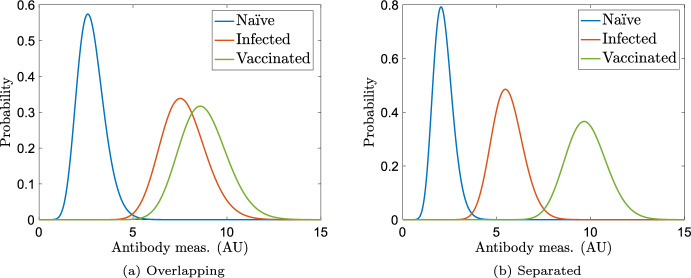
Distributions of antibody responses at time step 1 (21 days) for partially overlapping synthetic data motivated by Abela et al. (2021) and Congrave-Wilson et al. (2022) and synthetic data artificially generated to be more separated at $t = 21$ (Color Figure Online)

**Fig. 6:**
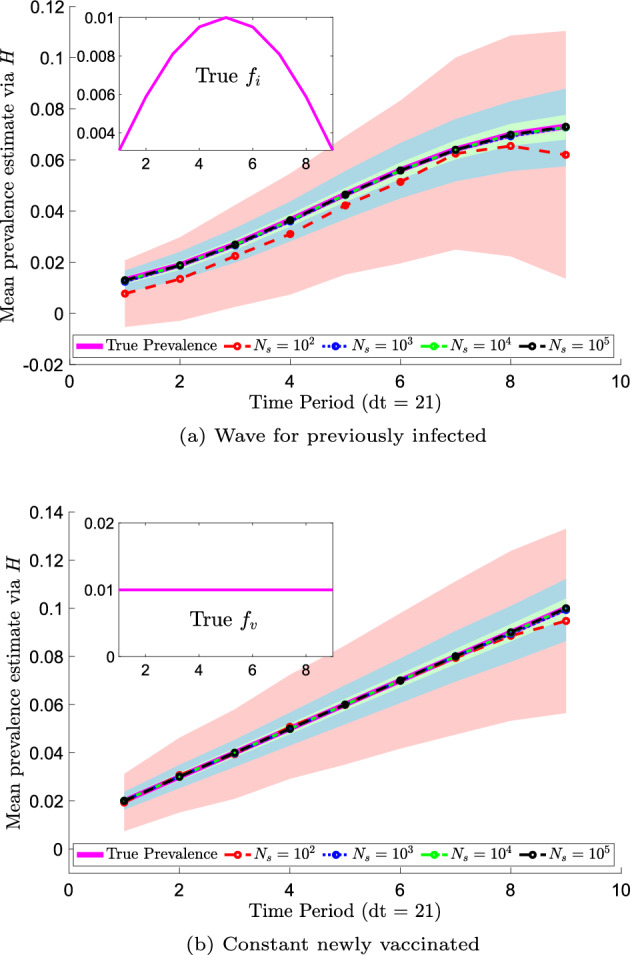
Prevalence estimation via transition probability estimates using synthetic data for antibody responses. The mean over 1000 synthetic data sets is shown for various numbers of samples $N_s$ with standard deviation confidence intervals (shown in a lighter shade of the corresponding color of $N_s$) over time (Color Figure Online)

We now revisit prevalence estimation via transition probability matrices after observing the large errors and standard deviations in our example shown in Fig. 4. A known issue in prevalence estimation and classification is overlap of class data (Luke et al. 2023). By plotting the probability models at one time step (21 days) in Fig. 5a, we note that the previously infected and vaccinated distributions exhibit significant overlap. As an exercise, we artificially create populations that exhibit more separation at the first time step using similar gamma distributions^3^, as shown at time step 1 in Fig. 5b. We conduct prevalence estimation via transition probability matrices for test data sampled from these distributions and display the results in Fig. 6. As before, prevalence estimation using the methods developed in Sect. 3.2 yields essentially identical results due to the equivalence in measurement densities, as proven in Sect. 4.3.1.

Note that a smaller number of samples, $N_s = 10^2$, is shown in Fig. 6 as compared to Fig. 4, and there are no negative prevalence estimates observed by using $N_s = 10^4$. Further, by using $N_s = 10^4$ samples, we are able to achieve average prevalence estimate errors across all time periods of $(5.6 \pm 4.6)$ % for infected and $(3.6 \pm 2.8)$ % for vaccinated, which is significantly better than the errors shown in Fig. 4 for $N_s = 10^5$. Using $N_s = 10^5$ for these better separated populations improves our errors to $(1.9 \pm 1.5)$ % for infected and $(1.2 \pm 0.9)$ % for vaccinated.

How many sample points are needed from the population at each time step might vary based on how separated the populations are, which can change over time. The importance of sampling may depend on the disease in question, which determines how separated the populations are naturally. We investigate the degree of separation over time of the distributions from the above example using the overlap metric defined by Weitzman (1970) as the shared area under two probability densities:

58 $$\begin{aligned} \text {OVL}(X,Y) = \int _{-\infty }^{\infty } \min \{ P_1(x), P_2(x) \} \ dx. \end{aligned}$$

**Fig. 7:**
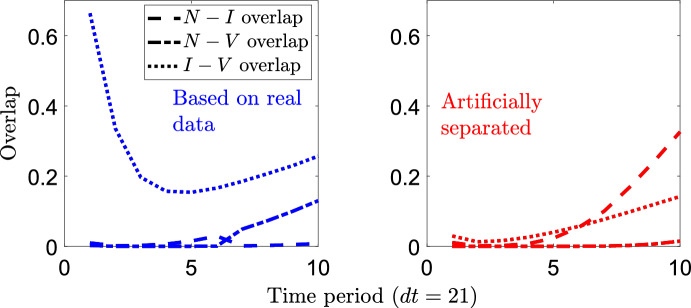
Measure of overlap of the antibody responses over time for the models that generated Fig. 5 (Color Figure Online)

We compare the pairwise overlap over time in Fig. 7. The overlapping infected and vaccinated distributions (Fig. 5a) agree on two-thirds of their underlying area at time step 1, whereas the artificially separated distributions have 3 % agreement at the same time. Interestingly, at time step 10 there is greater overlap of the infected and vaccinated populations for the artificially separated data; this suggests that separability is crucial in the early time steps due to the accumulation of errors. Schmid and Schmidt (2006) provide nonparametric estimators for the coefficient of overlap that could provide insight into the expected difficulty of prevalence estimation given sample populations with high levels of similarity. In future work, ideas from these authors could be extended to identify the number of samples needed to perform prevalence estimation with low variance.

For this synthetic example, we have demonstrated that better separation of populations in the first few time steps can result in more accurate prevalence estimation. We now discuss how to separate real data. First and foremost, if additional measurements per person are available, such as values for other protein markers, one can visualize the data in higher dimensions, thereby inducing separation (see e.g., Luke et al. 2023). If additional measurements are not available, there are ways to embed the data into a higher dimension, such as basis expansion from the field of kernel methods (Hastie et al. 2009). Metrics like the silhouette coefficient for data and the KL-divergence for distributions indicate a similarity score between populations, which we expect can determine if the populations are separated “enough” to conduct prevalence estimation. One can also borrow the idea of a holdout domain from Patrone et al. (2022) to exclude highly overlapped data from the prevalence estimation. Instead of a multiclass prevalence estimation, a two level binary procedure can be conducted: first to estimate the relative sizes of the naïve and not-naïve populations, and then another to separate the previously infected and vaccinated classes. For the secondary step, holdout analysis or other separation methods can be applied as needed. Finally, one can compute an altered estimate $\hat{\varvec{Q}}$ by summing indicator functions weighted inversely proportional to the overlap of the populations. This may lose the desirable property of unbiasedness, but we expect this to be overlooked in favor of reasonable prevalence estimates.

### Relationship to Susceptible–Infected–Recovered Models

Our approach is superior to an SIR framework, which can model disease transmission but cannot track the distribution of antibody response of a population across time. In contrast, we provide such probabilistic information as well as a prevalence estimation scheme that is independent of classification. However, there are connections between the two approaches. Probabilistic descriptions of SIR models exist, including Markov chain formulations (e.g., Liao et al. 2005; Cortés et al. 2020; El Hajji et al. 2021). Within-host SIR-like deterministic versions explicitly account for antibody densities and viral load as variables (Hancioglu et al. 2007). Our naïve state may be a proxy for the susceptible compartment, with a population-level equivalence of these two categories. We noted in Sect. 3.1 that unlike an SIR model, we have no recovered class; this leads to an interesting question. It is unclear how to mathematically describe the process by which someone becomes naïve after previous infection or vaccination. Our current framework assumes equivalence in the limit of time, but for a seasonal disease, there should be a nonzero probability of returning to *N* in finite time. We speculate that the naïve state is a recurrent state in finite time for the Markov chain model that allows transition back to naïve state.

### Limitations and Extensions

The choice of the form of the naïve model and shape functions for the infected and vaccinated populations are subjective choices (Smith 2013), but the influence of this issue is lessened as more sample points are used (Schwartz 1967). A family of models may be proposed and the one selected with minimal error on a measure of interest (Patrone and Kearsley 2024), such as prevalence estimates. As in our previous works, we have noted that the overlap of populations increases prevalence estimation error; we have provided potential solutions in Sect. 6.1. Additionally, Bedekar et al. (2022) first noted that prevalence estimation errors accumulate over time; our current scheme does not address controlling such errors.

Perhaps the largest drawbacks to our current approach are the simplifying assumptions we make to construct the groundwork for a multiclass time-dependent framework. For long-term analysis of disease emergence effects, multiple events must be allowed. In future work, we will relax the assumption that reinfection, revaccination, and infection after vaccination or vice versa do not occur. Mathematically, this involves a graph with higher connectivity, with edges connecting $\mathcal {I}'$ to $\mathcal {V}$, $\mathcal {I}'$ to $\mathcal {I}$, and so on. The many facets of the most general problem make tracking the immune response difficult, but we must consider such cases, because reinfections and revaccinations are the norm as a newly emergent disease becomes endemic. There is little biological understanding of interactions between such events and precious few models, as the antibody kinetics are still being studied. Modeling how these effects build on each other in terms of immune response is a complex question.

However, there are some guiding principles about immune memory from which to start building additive, probabilistic multi-event personal response models. Extreme sequences of events, such as an individual becoming newly infected every day, should be assigned very low probabilities due to the underlying biology. Further, despite the multitude of potential sequences of events leading to an antibody response and current state on a particular time step, the likelihood of infection or vaccination on the next time step depends solely on the current state information. Additionally, the multi-step transition probability matrix in the current Markov chain framework is considerably more structured than can be expected from the more general model. As a result, the estimation methods will need to be revised. These and other considerations inform our ongoing work to form a general model that can tackle real-life scenarios. As a first attempt at addressing such complexities, we are currently studying the setting in which we allow reinfection and revaccination, but simplify to a time-homogeneous Markov chain, which makes the calculation of desired quantities such as state probabilities more tractable. We will use this setting to inform the extension to the more complex time-inhomogeneous Markov chain.

There are significant data-related challenges that should be addressed in future work. One is simply a lack of temporal data, which affects the modeling process and prevalence estimation. Access to temporal random testing data from public health institutes can be used to verify the estimates by our models. Even when data are available, it can poorly represent the progression of an emerging disease through a population as truly random testing is not realistic. In the case of SARS-CoV-2, it is well known that people tested less frequently as the pandemic progressed, false negative tests occurred, and people often did not recognize mild or asymptomatic cases as infections. Furthermore, other confounders might cause skewed estimations of prevalence (Binder et al. 2024). Uncertainty quantification could address the deterioration of accuracy and precision of collected antibody data since the start of the pandemic. Prevalence estimation is a data-dependent analysis, which begins by estimating the incidence of new infections and vaccinations. Vaccination incidence rates may be well-documented for a population if de-identified medical data are available, but new infection case rates are prone to missing responses and errors due to the inexactness of days post symptom onset (DPSO) as an infection marker. Moreover, DPSO may often underestimate the true time since the beginning of infection. Future work could include the design of a prevalence estimation method that does not require knowing incidence information at the very emergence of a disease, with which we could verify our work using real incidence rates from a time period after all three of caseload numbers, vaccination rates, and antibody measurements are available. We note that immunocompromised individuals affect prevalence estimation and the modeling exercise. Future work could address the propagation of error by separating the population by immune system status, and correct errors due to reporting bias or gaps. We could also consider inflammatory markers as candidate variables, which have been studied in the context of severity of diseases such as the coronavirus disease of 2019 (COVID-19) (Zeng et al. 2020), but may not be fully understood in connection with sequences of immune response events.

### Implications for Immunologists

We have created a cohesive framework for the multiclass time-dependent problem of the emergence of a disease, so that unbiased predictions of the relative fractions of naïve, infected, and vaccinated individuals can be generated over time. Although we use SARS-CoV-2 as a motivating example, this approach is fully generalizable to other diseases for which immunity is lost on the time frame of months or a few years. In particular, the models follow biological assumptions that can be adapted or narrowed to focus on populations of interest, such as children, the elderly, or the immunocompromised. Our methods are limited by data availability; we recommend implementing longitudinal studies that continue to record infections with high granularity even as vaccines are deployed. As assay standardization is not fully achieved, such studies should use the same data collection methods, instruments, and protocol to facilitate the comparison of measurements across large periods of time.

## Data Availability

Analysis scripts and data developed as a part of this work are available upon reasonable request. Motivating data are provided in Abela et al. (2021) and Congrave-Wilson et al. (2022).

## Unbiasedness of the Prevalence Estimators

A straightforward extension of the ideas in Bedekar et al. (2022) shows that the prevalence estimates presented in Sect. 3.2 are unbiased. In what follows we continue to use element-wise vector addition to simplify our notation. First, it is clear that $\hat{\varvec{Q}}(T)$ is unbiased because each component is a Monte Carlo estimator and therefore

A1 $$\begin{aligned} \hat{Q}_j(T) \sim \frac{1}{S} \text {Binomial}(S, Q_j(T)), \end{aligned}$$

which implies

A2 $$\begin{aligned} E\left[ \hat{Q}_j(T) \right] = \frac{1}{S} E \left[ \text {Binomial}(S, Q_j(T))\right] = Q_j(T). \end{aligned}$$

Next, we find that

A3 $$\begin{aligned} E \left[ \hat{\varvec{f}}(0) \right] = E \left\{ [\varvec{M}(1) - \varvec{N}^*]^{-1} [\varvec{\hat{Q}}(1) - \varvec{N}]\right\} = [\varvec{M}(1) - \varvec{N}^*]^{-1} [E \{\varvec{\hat{Q}}(1) \} - \varvec{N}],\nonumber \\ \end{aligned}$$

and using the previous result we conclude that $E \left[ \hat{\varvec{f}}(0) \right] = \varvec{f}(0)$. Then, via induction, we find that

A4 $$\begin{aligned} E \left[ \hat{\varvec{f}}(T-1) \right]= &  E \left\{ [\varvec{M}(1) - \varvec{N}^*]^{-1} \left[ \varvec{\hat{Q}}(T) - \varvec{N} - \sum _{t = 0}^{T-2} [\varvec{M}(T-t) - \varvec{N}^*] \varvec{\hat{f}}(t) \right] \right\} \nonumber \\= &  [\varvec{M}(1) - \varvec{N}^*]^{-1} \left[ E \left[ \varvec{\hat{Q}}(T) \right] - \varvec{N} - \sum _{t = 0}^{T-2} [\varvec{M}(T-t) - \varvec{N}^*] E \left[ \varvec{\hat{f}}(t) \right] \right] \nonumber \\= &  [\varvec{M}(1) - \varvec{N}^*]^{-1} \left[ \varvec{Q}(T) - \varvec{N} - \sum _{t = 0}^{T-2} [\varvec{M}(T-t) - \varvec{N}^*] \varvec{f}(t) \right] \nonumber \\= &  \varvec{f}(T-1). \end{aligned}$$

Finally, we find that

A5 $$\begin{aligned} E \left[ \hat{\varvec{q}}(T) \right] = E \left[ \sum _{t = 0}^T \hat{\varvec{f}}(t) \right] = \sum _{t = 0}^T E \left[ \varvec{\hat{f}}(t) \right] = \sum _{t = 0}^T \varvec{f}(t) = \varvec{q}(T). \end{aligned}$$

## Unbiasedness of Transition Probability Estimation

The proof of the unbiasedness of the transition probability matrix entries can be obtained easily by using similar techniques as in Appendix A. We use linearity of expectation of the product of a deterministic matrix and a random vector on Eq. (45) to obtain

B1 $$\begin{aligned} \begin{aligned} E\left( \begin{bmatrix} \widehat{q}_N(0) \\ \widehat{H}_{0,(3,1)}\\ \widehat{H}_{0,(5,1)} \end{bmatrix}\right)&= E\left( \begin{bmatrix} N_{D_1} & R_{D_1}(1) & W_{D_1}(1)\\ N_{D_2} & R_{D_2}(1) & W_{D_2}(1)\\ 1 & 1 & 1 \end{bmatrix} ^{-1}\begin{bmatrix} \widehat{Q}_{D_1}(1) \\ \widehat{Q}_{D_2}(1)\\ 1 \end{bmatrix} \right) \\&= \begin{bmatrix} N_{D_1} & R_{D_1}(1) & W_{D_1}(1)\\ N_{D_2} & R_{D_2}(1) & W_{D_2}(1)\\ 1 & 1 & 1 \end{bmatrix} ^{-1}E\left( \begin{bmatrix} \widehat{Q}_{D_1}(1) \\ \widehat{Q}_{D_2}(1)\\ 1 \end{bmatrix} \right) . \end{aligned} \end{aligned}$$

Using the unbiasedness of the Monte Carlo estimates as shown in Eqs. (A1) and (45) again, we get

B2 $$\begin{aligned} E\left( \begin{bmatrix} \widehat{q}_N(0) \\ \widehat{H}_{0,(3,1)}\\ \widehat{H}_{0,(5,1)} \end{bmatrix}\right) = \begin{bmatrix} N_{D_1} & R_{D_1}(1) & W_{D_1}(1)\\ N_{D_2} & R_{D_2}(1) & W_{D_2}(1)\\ 1 & 1 & 1 \end{bmatrix} ^{-1} \begin{bmatrix} Q_{D_1}(1) \\ Q_{D_2}(1)\\ 1 \end{bmatrix} = \begin{bmatrix} q_N(0) \\ H_{0,(3,1)}\\ H_{0,(5,1)} \end{bmatrix}. \end{aligned}$$

These calculations are easily generalized by using the principle of strong induction and Eqs. (46), (A1), and (B2), to obtain

B3 $$\begin{aligned} &  E\left( \begin{bmatrix} \widehat{q}_N(T-1) \\ \widehat{H}_{(T-1)(3,1)}\\ \widehat{H}_{(T-1)(5,1)} \end{bmatrix}\right) \nonumber \\ &  \quad = \begin{bmatrix} N_{D_1} & R_{D_1}(1) & W_{D_1}(1)\\ N_{D_2} & R_{D_2}(1) & W_{D_2}(1)\\ 1 & 1 & 1 \end{bmatrix} ^{-1} \nonumber \\ &  \quad \times E\left( \begin{bmatrix} \widehat{Q}_{D_1}(T) - \sum \nolimits _{t=0}^{T-2} \left( R_{D_1}(T-t) \widehat{H}_{t,(3,1)} + W_{D_1}(T-t) \widehat{H}_{t,(5,1)} \right) \\ \widehat{Q}_{D_2}(T) - \sum \nolimits _{t=0}^{k-2} \left( R_{D_2}(T-t) \widehat{H}_{t,(3,1)} + W_{D_2}(T-t) \widehat{H}_{t,(5,1)} \right) \\ \widehat{q}_N(T-2) \end{bmatrix}\right) \nonumber \\ &  \quad = \begin{bmatrix} N_{D_1} & R_{D_1}(1) & W_{D_1}(1)\\ N_{D_2} & R_{D_2}(1) & W_{D_2}(1)\\ 1 & 1 & 1 \end{bmatrix}^{-1} \nonumber \\ &  \quad \times \begin{bmatrix} Q_{D_1}(T) - {\sum \nolimits _{t=0}^{T-2}} \left( R_{D_1}(T-t) H_{t,(3,1)} + W_{D_1}(T-t) H_{t,(5,1)} \right) \\ Q_{D_2}(T) - \sum \nolimits _{t=0}^{T-2} \left( R_{D_2}(T-t) H_{t,(3,1)} + W_{D_2}(T-t) H_{t,(5,1)} \right) \\ q_N(T-2) \end{bmatrix} \nonumber \\ &  \quad = \begin{bmatrix} q_N(T-1) \\ H_{(T-1)(3,1)}\\ H_{(T-1)(5,1)} \end{bmatrix}. \end{aligned}$$

The estimates for the other entries of the transition probability matrix are also unbiased. Using Eqs. (47) and (48) we see that

B4 $$\begin{aligned} E\left( \widehat{H}_{(T-1)(2,1)}\right) = \sum \limits _{\tau =0}^{T-2} E\left( \widehat{H}_{\tau (3,1)}\right) = \sum \limits _{\tau =0}^{T-2} H_{\tau (3,1)} = H_{(T-1)(2,1)}. \end{aligned}$$

and

B5 $$\begin{aligned} E\left( \widehat{H}_{(T-1)(4,1)}\right) = \sum \limits _{\tau =0}^{T-2} E\left( \widehat{H}_{\tau (5,1)}\right) = \sum \limits _{\tau =0}^{T-2} H_{\tau (5,1)} = H_{(T-1)(4,1)}. \end{aligned}$$

## Optimal Classification

Optimal classification can be performed to yield class domains that vary over time. Since the antibody kinetics of states *N* and $\mathcal {I}$ share the same distribution, newly infected individuals cannot be distinguished from those naïve to the disease. This reflects the known delay in antibody response after infection (Borremans et al. 2020). Thus, antibody measurements are classified as belonging to one of *N*, $\mathcal {I}'$, or $\mathcal {V}'$.

We combine the ideas in Bedekar et al. (2022) and Luke et al. (2023) to construct the optimal classification domains that minimize a loss function. We seek to define a sequence of partitions $\{D_N(T), D_{\mathcal {I}'}(T), D_{\mathcal {V}'}(T)\}$, not necessarily the same as any partition used for prevalence estimation, so that at any time *T*, a measurement is assigned to one and only one class, i.e., *N* if $\varvec{r} \in D_N(T)$, and similarly for $D_{\mathcal {I}'}(T)$ and $D_{\mathcal {V}'}(T)$. To ensure any sample can be classified and to enforce single-label classification up to sets of measure zero, we require, for any *T*, that

C1 $$\begin{aligned} &  \mu _N \left( \cup _D \right) = \mu _{\mathcal {I}'} \left( \cup _D \right) = \mu _{\mathcal {V}'} \left( \cup _D \right) = 1, \end{aligned}$$

C2 $$\begin{aligned} &  \mu _{J} [D_{L}(T) \cap D_K(T) ] = 0 \text { for } L \ne K, \text { for } J \in \{N, \mathcal {I}', \mathcal {V}'\}. \end{aligned}$$

Here, $\cup _D = D_N(T) \cup D_{\mathcal {I}'}(T) \cup D_{\mathcal {V}'}(T)$, the subscripts *L* and *K* can represent any of $N, \mathcal {I}'$, or $\mathcal {V}'$, and $\mu _N(X) = \int _X N(\varvec{r}) d \varvec{r}$, $\mu _{\mathcal {I}'}(X) = \int _X \mathcal {I}'(\varvec{r}, T) d \varvec{r}$, and $\mu _{\mathcal {V}'}(X) = \int _X \mathcal {V}'(\varvec{r}, T) d \varvec{r}$.

Next, define the prevalence-weighted rate of misclassification at time *T*:

C3 $$\begin{aligned} \begin{aligned} \mathscr {L}\left( D_N(T), D_{\mathcal {I}'}(T), D_{\mathcal {V}'}(T) \right)&= q_N(T) \int _{\Omega \setminus D_N(T)} N(\varvec{r}) d \varvec{r} \\&+ q_{\mathcal {I}'}(T) \int _{\Omega \setminus D_{\mathcal {I}'}(T)} \mathcal {I}'(\varvec{r}, T) d \varvec{r} \\&+ q_{\mathcal {V}'}(T) \int _{\Omega \setminus D_{\mathcal {V}'}(T)} \mathcal {V}'(\varvec{r}, T) d \varvec{r}. \end{aligned} \end{aligned}$$

One expects that a sample $\varvec{r}$ should be assigned to the class to which it has the highest probability of belonging on time step *T*; that is, $\max \{ q_N(T) N(\varvec{r}), q_{\mathcal {I}'}(T) \mathcal {I}'(\varvec{r}, T), q_{\mathcal {V}'}(T) \mathcal {V}'(\varvec{r}, T) \}$. It turns out that our intuition is correct. The authors in Luke et al. (2023) showed that for a single time *T*, the optimal classification domains are given by

C4 $$\begin{aligned} \begin{aligned} D_N^{\star }(T)&= \{ \varvec{r}: q_N(T) N(\varvec{r})> q_{\mathcal {I}'}(T) \mathcal {I}'(\varvec{r}, T) \} \\&\qquad \cap \{\varvec{r}: q_N(T) N(\varvec{r})> q_{\mathcal {V}'}(T) \mathcal {V}'(\varvec{r},T)\}, \\ D_{\mathcal {I}'}^{\star }(T)&= \{ \varvec{r}: q_{\mathcal {I}'}(T) \mathcal {I}'(\varvec{r}, T)> q_N(T) N(\varvec{r}) \} \\&\qquad \cap \{\varvec{r}: q_{\mathcal {I}'}(T) \mathcal {I}'(\varvec{r}, T)> q_{\mathcal {V}'}(T) \mathcal {V}'(\varvec{r},T)\}, \\ D_{\mathcal {V}'}^{\star }(T)&= \{ \varvec{r}: q_{\mathcal {V}'}(T) \mathcal {V}'(\varvec{r}, T)> q_N(T) N(\varvec{r}) \} \\&\qquad \cap \{\varvec{r}: q_{\mathcal {V}'}(T) \mathcal {V}'(\varvec{r}, T) > q_{\mathcal {I}'}(T) \mathcal {I}'(\varvec{r},T)\}. \end{aligned} \end{aligned}$$

This assumes a technical detail that all “boundary” cases, i.e., measurements $\varvec{r}$ such that there is an equal highest probability of belonging to two or more classes, have measure zero. Since this is often true for any practical implementation, we will assume this going forward. Also note that the use of a superscript $\star$ denotes an optimal quantity, in this case, an optimal classification domain.

A logical loss function is then the sum of these rates over all time steps of the emergence of the disease:

C5 $$\begin{aligned} \mathscr {L}_{\tau } (\varvec{D}_{\varvec{N}}, \varvec{D}_{\varvec{\mathcal {I}'}}, \varvec{D}_{\varvec{\mathcal {V}'}}) = \sum _{T = 0}^{\tau } \mathscr {L}(D_N(T), D_{\mathcal {I}'}(T), D_{\mathcal {V}'}(T)), \end{aligned}$$

where each of $D_N(T), D_{\mathcal {I}'}(T), D_{\mathcal {V}'}(T)$ obey the rules given by Eqs. (C1)-(C2) and $\varvec{D}_{\varvec{N}}$ is a vector whose *i*th entry is $D_N(i)$. The vectors $\varvec{D}_{\varvec{\mathcal {I}'}}$ and $\varvec{D}_{\varvec{\mathcal {V}'}}$ are defined analogously. A straightforward application of the ideas in Bedekar et al. (2022) shows us that the loss function (C5) is minimized by taking the pointwise-optimal domains at each time step *T* given by Eq. (C4). Thus, the optimal classification domains are given by the vectors

C6 $$\begin{aligned} \varvec{D}_{\varvec{N}}^{\star } = \begin{bmatrix} D_N^{\star }(0) \\ D_N^{\star }(1) \\ \vdots \\ D_N^{\star }(\tau ) \end{bmatrix}, \quad \varvec{D}_{\varvec{\mathcal {I}'}}^{\star } = \begin{bmatrix} D_{\mathcal {I}'}^{\star }(0) \\ D_{\mathcal {I}'}^{\star }(1) \\ \vdots \\ D_{\mathcal {I}'}^{\star }(\tau ) \end{bmatrix}, \quad \varvec{D}_{\varvec{\mathcal {V}'}}^{\star } = \begin{bmatrix} D_{\mathcal {V}'}^{\star }(0) \\ D_{\mathcal {V}'}^{\star }(1) \\ \vdots \\ D_{\mathcal {V}'}^{\star }(\tau ) \end{bmatrix}. \end{aligned}$$

## Footnotes

1 Certain commercial equipment, instruments, software, or material are identified in this paper in order to specify the experimental procedure adequately. Such identification is not intended to imply recommendation or endorsement by the National Institute of Standards and Technology, nor is it intended to imply that the materials or equipment identified are necessarily the best available for the purpose.

2 This assumption holds for an emergent disease; we would expect almost none of the population to be in the naïve state in the case of an endemic virus such as the common cold.

3 The model parameters are $\alpha _n = 17.6, \beta _n = 0.123$, $\theta _{1, i} = 2.23, \theta _{2, i} = 5.3 \times 10^{-4}, \theta _{1, v} = 4.23$, and $\theta _{2, v} = 5.3 \times 10^{-4}$.
